# High-efficiency exfoliation of layered materials into 2D nanosheets in switchable CO_2_/Surfactant/H_2_O system

**DOI:** 10.1038/srep16764

**Published:** 2015-11-16

**Authors:** Nan Wang, Qun Xu, Shanshan Xu, Yuhang Qi, Meng Chen, Hongxiang Li, Buxing Han

**Affiliations:** 1 College of Materials Science and Engineering, Zhengzhou University, Zhengzhou 450052, China; 2 Institute of Chemistry, Chinese Academy of Science, Beijing 100080, China

## Abstract

Layered materials present attractive and important properties due to their two-dimensional (2D) structure, allowing potential applications including electronics, optoelectronics, and catalysis. However, fully exploiting the outstanding properties will require a method for their efficient exfoliation. Here we present that a series of layered materials can be successfully exfoliated into single- and few-layer nanosheets using the driving forces coming from the phase inversion, i.e., from micelles to reverse micelles in the emulsion microenvironment built by supercritical carbon dioxide (SC CO_2_). The effect of variable experimental parameters including CO_2_ pressure, ethanol/water ratio, and initial concentration of bulk materials on the exfoliation yield have been investigated. Moreover, we demonstrate that the exfoliated 2D nanosheets have their worthwhile applications, for example, graphene can be used to prepare conductive paper, MoS_2_ can be used as fluorescent label to perform cellular labelling, and BN can effectively reinforce polymers leading to the promising mechanical properties.

Following the advent of graphene in 2004, it was soon recognized that in addition to the composition and arrangement of atoms in materials, dimensionality plays a critical role in determining their fundamental properties^1^,^2^. Over the past decade, some significant developments have been spearheaded by research into two-dimensional (2D) layered nanomaterials considering that they have potential applications in a wide range of fields such as electronics, bio-sensors, catalysis, and energy storage, etc^3^,^4^,^5^,^6^,^7^,^8^,^9^,^10^,^11^,^12^,^13^. Obviously, successful scale-up fabrication of high-quality layered materials is the prerequisite for achieving their full potential^14^,^15^. Micromechanical cleavage is a simple method, but it is on a small scale and has disadvantages in commercial high-end applications^16^. Intercalation technique is a useful alternative top-down method to exfoliate layered materials by using various kinds of intercalates, such as alkali metals and transition-metal halides^17^,^18^,^19^,^20^. However, ion intercalation-based methods have drawbacks associated with their sensitivity to ambient conditions. Coleman and other researchers have made a major breakthrough via the sonication-assisted exfoliation in nonvolatile organic solvents or mixed-solvents, like dimethylsulphoxide (DMSO), N-methyl-pyrrolidinone (NMP), N-vinyl-Pyrrolidinone (NVP), to produce mono- and few-layer nanosheets^21^,^22^,^23^,^24^. It is a simple liquid exfoliation method following the mechanism that the surface energy of the solvent must match that of the solute, but the employment of nonvolatile organic solvents will impede further applications of layered nanomaterials and bring the negative impact to the environment.

Besides the methods referred above, surfactant-assisted exfoliation is of particular interest^25^,^26^,^27^. First, the used solvent is water and so it is benign to environment. Second, the application of surfactants caters for the exfoliation demand that it is necessary to enhance the ratio of surface to mass and to form larger interface. It is well-known that in a micelle, the hydrophobic tail of the surfactant points towards the core while the polar head group forms an outer shell. Similarly, surfactant may also aggregate in non-polar organic solvent, wherein the structure was referred as reverse micelles^28^. As an excellent and green alternative to conventional organic solvents^29^,^30^,^31^, supercritical carbon dioxide (SC CO_2_) possesses an important property that it can assist surfactant-water solutions to build the reverse-micelle emulsions microenvironment, and the phase behaviour of emulsions microenvironment can be manipulated by tuning the physical property of solution^32^,^33^,^34^. Specifically, just as a “switch” for the molecular aggregation of surfactants, the tuning of the aggregation behaviours of surfactants by CO_2_ is reversible, which can be realized by simply pressurization and depressurization^35^.

In this study, for the first time we present a highly facile, efficient and versatile method to exfoliate a series of layered materials by virtue of SC CO_2_ to switch the phase inversion from micelles to reverse micelles in the emulsion microenvironment. We have systematically explored the typical layered materials such as graphene, MoS_2_, WS_2_, and BN in the emulsions microenvironment of CO_2_/surfactant/H_2_O system, and obtained the detail information about optimal solution condition of their efficient exfoliation. Experimental results demonstrate that the driving force coming from the phase inversion from micelles to reverse micelles is efficient for the exfoliation, and as well as the curvature transition of surfactants and the phase behaviours of micelles in the emulsions can be manipulated by changing formulation variables, such as CO_2_ pressure and ethanol/water ratio^36^,^37^,^38^. Further the exfoliated graphene can be used to prepare high-conductive paper, MoS_2_ nanosheets can be used as broad-spectrum fluorescent label to perform cellular labelling, and BN nanosheets can be used to efficiently reinforce the mechanical properties of polymer. So this strategy utilizing reverse-micelle-induced method for exfoliation of layered materials has great potential application in electronic, biotechnology, mechanics, energy and information storage, etc.

## Results

### Production of layered materials

**Figure 1:**
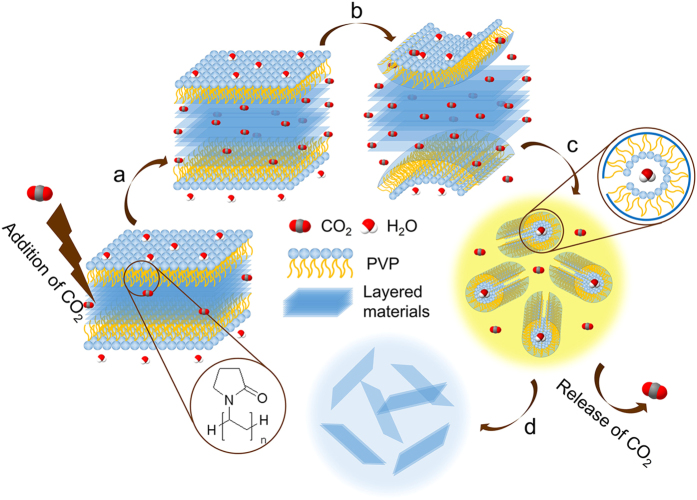
A schematic diagram of the exfoliation process of layered materials in the emulsion microenvironment of CO_2_/PVP/H_2_O system. (**a**) CO_2_ molecules impregnate into the interlayers of layered materials, weakening the ineractions between the adjacent interlayers. (**b,c**) The phase inversion of emulsions results in curvature transition of surfactants LM-PVP and the repulsive forces driving the curvature transition delaminate ultrathin 2D nanosheets from bulk materials. (**d**) The 2D nanosheets disperse stably in ethanol/water mixtures after CO_2_ is released.

Figure 1 shows the schematic diagram of the exfoliation process of layered materials in the emulsions microenvironment of the CO_2_/surfactant/H_2_O system. Since PVP can adsorb on the surface of these layered materials through the strong hydrophobic interactions between PVP chains and surface of layered materials^25^,^39^,^40^, we defined a conception that layered materials and PVP can combine into a whole, defined as the “block” surfactant LM-PVP (LM refer to layered materials). The hydrophilic amide groups of LM-PVP point toward the water continuous phase, whereas hydrophobic portions of LM-PVP including hydrophobic alkyl chains and the structure of layered materials exist in the interior of surfactants.

First, LM-PVP solution was transferred into SC CO_2_ system. At the lower CO_2_ pressures, gaseous CO_2_ can dissolve into continuous water phase and enter surfactant interfacial region on account of the interaction between CO_2_ and the surfactant, suggesting the formation of CO_2_-in-water emulsions. Second, with increasing CO_2_ pressure or density, more CO_2_ molecules penetrated into the interlayers of layered materials with high diffusivity (Fig. 1a), contributing to the expansion of the distance between adjacent layers and thus the decrease in the interaction between them^41^. Driven by minimization of the interfacial free energy of emulsions (Supplementary Fig. S9 and Supplementary Note 2), surfactant LM-PVP rolled up and formed a tube via the strong repulsive forces between hydrophilic groups and CO_2_ molecules, resulting in the curvature transition of LM-PVP^42^ (Fig. 1b,c). Meanwhile, single- or few-layered 2D nanosheets were delaminated from bulk materials via curvature transition of surfactant drived by the repulsive forces. Finally, after CO_2_ was released, water-in-CO_2_ emulsions containing reverse micelles transformed into normal micelle solution with water as a continuous phase, and a large number of individual 2D nanosheets were prepared and dispersed stably in solution due to steric stabilization of PVP (Fig. 1d).

### Materials characterization

**Figure 2:**
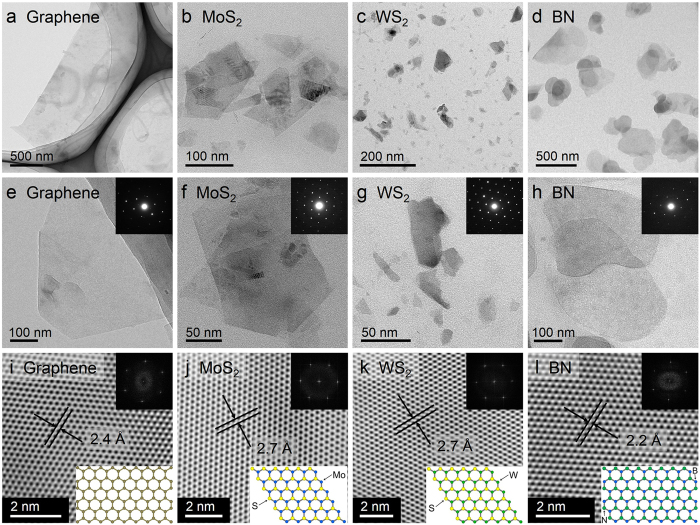
Mophology of exfoliated 2D nanosheets and their atomic structure. (**a**–**d**) Low-magnification TEM images of flakes of graphene, MoS_2_, WS_2_, and BN, respectively. (**e–h**) High-magnification TEM images of flakes of graphene, MoS_2_, WS_2_, and BN, respectively. (**i–l**) High-resolution TEM (HRTEM) images of flakes of graphene, MoS_2_, WS_2_, and BN nanosheets, respectively. (**Insets**) Top: Fast Fourier transforms of the images; Bottom: Schematic drawing of the atomic structure of graphene, MoS_2_, WS_2_, and BN, respectively.

We exfoliated a series of layer materials of graphene, MoS_2_, WS_2_, and BN in the emulsions microenvironment of the CO_2_/PVP/H_2_O system (Supplementary Fig. S1). Transmission electron microscopy (TEM) examination of materials deposited from the dispersions shows the existence of large quantities of ultrathin 2D sheets (Fig. 2a–d). The ranges of lateral size of graphene, MoS_2_, WS_2_, and BN are different possibly due to the different size of their bulk materials. Figures 2e–h show that these 2D nanosheets turn slightly transparent to the electron beam due to their ultrathin structure. The diffraction patterns in Fig. 2e–h illustrate typical six-fold symmetry of highly crystalline structure. Additional TEM micrographs of these nanosheets are shown in Supplementary Fig. S3. Figure 2i–l are high-resolution TEM micrographs (HRTEM) of graphene, MoS_2_, WS_2_, and BN, providing more detailed structural information. The integrity and uniformity of the lattice structure of these nanosheets suggest that defects and deformation were not introduced in the fabrication process. These images reveal graphene lattice spacing of 2.4 Å, MoS_2_ and WS_2_ lattice spacing of 2.7 Å, and BN lattice spacing of 2.2 Å. Addtionally, the analysis of HRTEM intensity profiles suggests the presence of monolayer in the dispersions^21^,^43^. A near perfect planar structure of monolayer 2 H-MoS_2_ without defects and deformation is shown in Supplementary Fig. S4. A significant variation in intensity between neighboring atom peaks can be observed from the intensity profile across the red dashed line in Supplementary Fig. S4c. For ABAB stacking order of the 2 H-MoS_2_, there is no difference in the micrograph intensity between neighboring atom peaks if the nanosheets are more than two layers^43^. Therefore, the significant variation in intensity suggests the exfoliated MoS_2_ nanosheet to be the planar 2D structure with monolayer.

**Figure 3:**
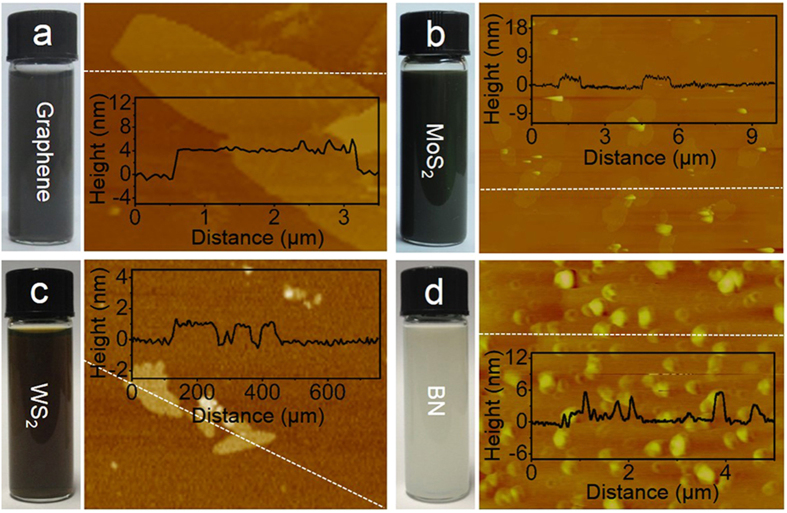
The thickness and dimension of exfoliated 2D nanosheets. (**a–d**) The dispersions of graphene, MoS_2_, WS_2_, and BN nanosheets and their AFM images respectively. Inset: thickness profiles along the white lines shown in the AFM images (**a–d**), respectively.

Further examination of the nature of the nanosheets was performed by atomic force microscopy (AFM) measurement (Fig. 3), which presents the thickness of exfoliated 2D materials. The MoS_2_ nanosheets have different thicknesses with the majority in the range of 2–5 nm. Since the thicknesses of the single-layer MoS_2_ nanosheets were determined to be in the range of 0.9–1.2 nm^23^, AFM measurement confirms that the exfoliated MoS_2_ nanosheets consist of 2–4 monolayers. Similarly, the thicknesses of graphene (3–5 nm), WS_2_ (1–3 nm), and BN (2–6 nm) nanosheets indicate that they also exist as few-layer nanosheets in dispersions. The absorption spectra of exfoliated 2D nanosheets dispersions is illustrated in Supplementary Fig. S5. These characteristic absorption curves ranging from 300 to 900 nm coincide with the general features of exfoliated 2D layered materials^21^,^22^. The Raman spectra of the exfoliated MoS_2_ nanosheets were recorded using a 514 nm excitation line. As shown in Supplementary Fig. S6, the smaller frequency difference (Δ) between E^1^_2g_ and A_1g_ modes for the few-layer MoS_2_ (Δ = 24.7 cm^−1^) in comparison with that of the bulk MoS_2_ (Δ = 26.9 cm^−1^) manifests the significant reduction of the sheets thicknesses from the bulk MoS_2_ to the exfoliated samples^44^. According to the “Δ-thickness relation” established by previous works based on exfoliated samples, the thicknesses of exfoliated 2D MoS_2_ nanosheets are mostly less than 5 monolayers (<4 nm). From XPS analysis, the exfoliated MoS_2_ nanosheets show Mo *3d* peaks with peak position and width characteristic of the 2 H phase^45^ (Supplementary Fig. S7).

### Effect of CO_2_ pressure, ethanol/water ratio and initial concentration of bulk materials

**Figure 4:**
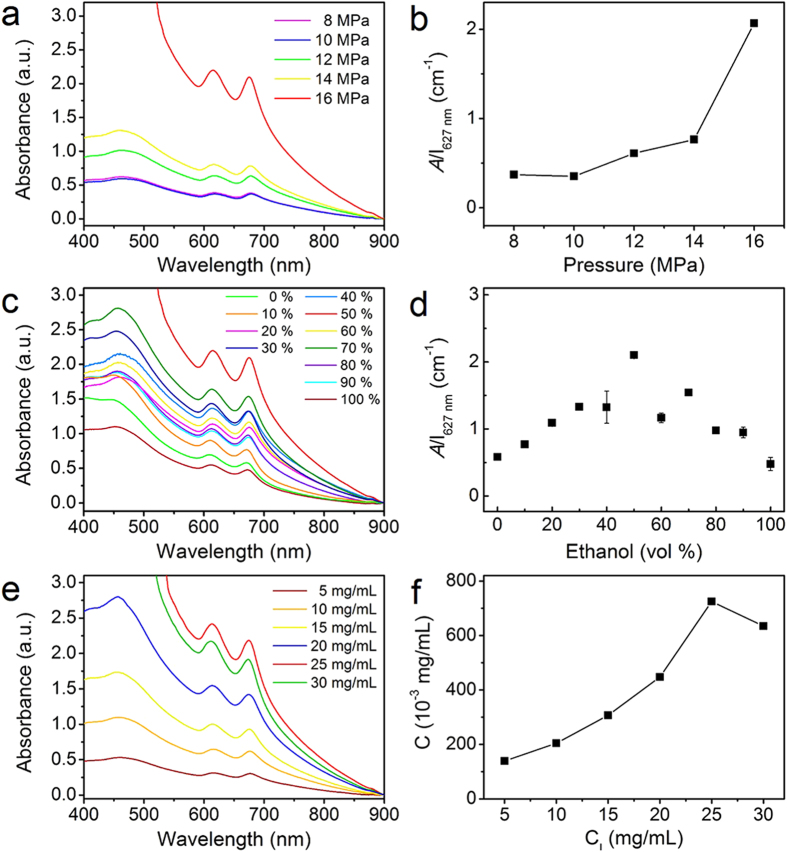
Effect of CO_2_ pressure, ethanol/water ratio and initial concentration of bulk materials on the exfoliation yield. (**a**) Absorption spectra of MoS_2_ dispersed in ethanol/water mixtures with ratio of 1:1 at 313.2 K and different CO_2_ pressures. (**b**) Absorbance of MoS_2_ dispersed in ethanol/water mixtures with ratio of 1:1 at 313.2 K and different CO_2_ pressures. (**c**) Absorption spectra of MoS_2_ dispersed in ethanol/water mixtures with different ratios. (**d**) Absorbance of MoS_2_ dispersed in ethanol/water mixtures with different ratios. (**e**) Absorption spectra of MoS_2_ dispersed in ethanol/water mixtures with ratio of 1:1 at different MoS_2_ initial concentrations. (**f**) Absorbance of MoS_2_ dispersed in ethanol/water mixtures with ratio of 1:1 at different MoS_2_ initial concentrations.

By analysis of the phase behaviours of emulsions of the CO_2_/PVP/H_2_O system (Supplementary Fig. S8 and Supplementary Note 1), we found that CO_2_ pressure has a significant effect on the phase behaviour of emulsions. Therefore, we investigated the effect of CO_2_ pressure on the exfoliation yield of layered materials (Fig. 4a,b). Take MoS_2_ as a typical example, as expected, little exfoliation was detected at the CO_2_ pressure ranging from 8 to 10 MPa. With increasing CO_2_ pressure to 12 MPa, the absorbance of as-fabricated MoS_2_ dispersions increased, which is attributed to the occurrence of curvature transition of surfactant drived from the phase inversion of emulsions at 12 MPa or higher pressure^42^.

We also investigated the effect of ethanol/water ratio on the exfoliation yield of layered materials (Fig. 4c,d and Supplementary Fig. S10,S11). Apparently there was slight exfoliation occurred for MoS_2_ in pure water. Similarly, little exfoliation was observed in pure ethanol. Whereas, an appropriate ethanol/water ratio resulted in the obvious exfoliation of MoS_2_. For MoS_2_, the most effective exfoliation appeared with an ethanol content around 50 vol%. Given that ethanol is highly soluble in CO_2_, the addition of ethanol as cosolvent may be expected to improve the polarity of SC CO_2_, which is favorable to promote the solubility of surfactant in SC CO_2_. Moreover, with the addition of ethanol, the interactions (*A*_*TT*_) between the surfactant tails become weaker, which leads to increase of 1/HCB and decrease of interfacial tension and interfacial tension gradients, and thus emulsions tend to be unstable, resulting in the phase inversion from CO_2_-in-water emulsions to water-in-CO_2_ emulsions. In the experiments, we found that the concentration of MoS_2_ and graphene dispersions both were strongly related to ethanol/water ratio. It is likely that the synergistic effect of water and ethanol has a great effect on the phase inversion of emulsions and results in the different exfoliation efficiency. A series of measured concentration values of MoS_2_ dispersions with ethanol content from 0 to 100 vol% are shown in Supplementary Table S1. The highest concentration was calculated to be approximately 0.139 mg/mL, which is higher than those concentrations reported^23^. In addition, we explored the relationship between exfoliated MoS_2_ concentration (C) and initial MoS_2_ concentration (C_I_) (Fig. 4e,f and Supplementary Fig. S13). Figure 4e shows the concentration of MoS_2_ dispersions fabricated at different initial MoS_2_ concentration of 5 to 30 mg/mL. It clearly appears that the exfoliated MoS_2_ concentration increased significantly with increasing initial MoS_2_ concentration. At initial MoS_2_ concentration of 25 mg/mL, the exfoliated MoS_2_ concentration dispersions reached the highest value, which is approximately 0.725 mg/mL.

### Potential applications of as-exfoliated 2D nanosheets

**Figure 5:**
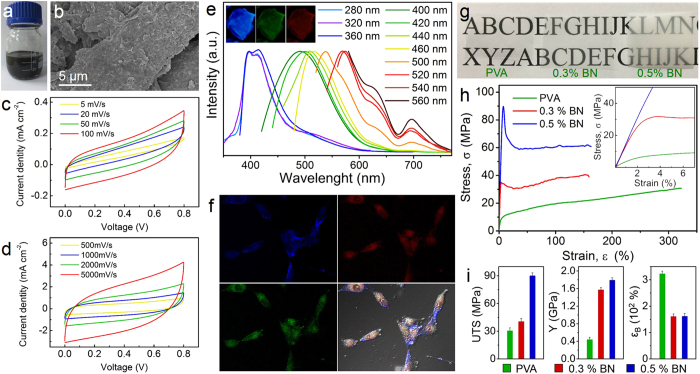
Potential applications of 2D nanosheets in electronics, biology and mechanical reinforcement. (**a**) Photographs of as-fabricated graphene dispersions. (**b**) The SEM image of the films obtained by brushing graphene nanosheets on the paper. (**c**) CV curves of graphene at different scan rates of 5, 20, 50 and 100 mV s^−1^. (**d**) CV curves of graphene at different scan rates of 500, 1000, 2000 and 5000 mV s^−1^. (**e**) PL spectra of MoS_2_ nanosheets under excitation wavelengths of 280–560 nm. Inset is the fluorescence photographs of MoS_2_ sheets under the excitation of ultraviolet, blue, and green lasers lines respectively. (**f**) Fluorescent images of lung cancer cells stained with MoS_2_ nanosheets at broadband excitation light sources of UV, blue, and green. (**g**) Photograph showing a pure PVA film (left) and free-standing composite films filled with 0.3 wt% (middle) and 0.5 wt% (right) BN nasheets. (**h**) Representative stress-strain curves for composites of polyvinyl alcohol (PVA) filled with BN at loading levels of 0  wt% and 0.5 wt%. (**i**) Young’s modulus, ultimate tensile strength, and strain to break for the PVA reference and composites of polyvinyl alcohol (PVA) filled with BN nanosheets.

The successful exfoliation of these layed materials bring us more possibilities in application. To examine the electronic properties of as-prepared graphene nanosheets, a dispersible graphene “ink” was prepared by dispersing graphene in DMF with a concentration of 10 mg mL^−1^, and highly conductive graphene films were readily fabricated by brushing the as-prepared graphene ink on commercial paper (Supplementary Note 3). The electrochemical performance of the electrode materials was investigated by cyclic voltammetry (Fig. 5c,d). This binder- and additive-free graphene paper-based supercapacitor presented typical double-layer capacitive behaviours at different scanning rate. The area capacitance of the flexible supercapacitor is 11.1 mF cm^−2^ at the scanning rate of 5 mV s^−1^, which is much superior to that of graphene fabricated by electrochemical exfoliation (11.3 mF cm^−2^ at a low scan rate of 1 mV s^−1^)^42^. Remarkably, the electrode materials were found to exhibit an excellent rectangular mirror image even at the scanning rate of 5000 mV s^−1^, indicating an outstanding rate capability and electrical performance. Besides, with a graphene loading of 0.682 mg cm^−2^, a sheet resistance of the graphene paper reduced to 2.41Ω sq^−1^, which is much better than that of rGO films (43 KΩ sq^−1^) and graphene exfoliated by electrochemical exfoliation (11 Ω sq^−1^)^46^,^47^. Therefore, it confirms that our exfoliation method retains the integrity of the perfect structure of graphene to the largest extent, thereby producing graphene’s remarkable electrical properties.

The photoluminescence (PL) spectra obtained at various excitation wavelengths ranging from 280 to 560 nm provide the PL properties of MoS_2_ nanosheets (Fig. 5e). It was observed a red shift in the PL spectra of the nanosheets over emission wavelengths ranging from 396 to 580 nm. For the excitation wavelength in the range of 500 to 560 nm, besides the strong emission peak, two weak shoulder emission appeared at 625 nm and 695 nm. In addition, by analysis of fluorescence microscope, the corresponding phenomena were observed in the dried MoS_2_ that exhibit the blue, green, and red emission under the excitation of the light sources of UV, blue, and green, respectively (Inset in Fig. 5e). The extraordinary PL properties of the as-exfoliated MoS_2_ propel us to use it as fluorescent label to perform cellular labelling^48^,^49^, and the experiment was performed on lung cancer cells. Before labeling, cells were incubated with MoS_2_ nanosheets at 37 °C for 24 h. More detailed preparation methods was shown in the Supplementary Note 4. In the lung cancer cells stained with MoS_2_, the nanosheets were taken up by the cells and agglomerated in the cells. Figure 5f shows the fluorescent images of lung cancer cells stained with MoS_2_ nanosheets at broadband excitation light sources of UV (300–400 nm), blue (400–500 nm), and green (500–600 nm), exhibiting the blue, green, and red emission, respectively.

The exfoliated BN nanosheets were found to be quite useful for reinforcement of polymeric films. A stable and environmentally robust dispersion allows BN nanosheets as excellent fillers to prepare high performance composites of polyvinyl alcohol (PVA) (Fig. 5g and Supplementary Note 5). Representative stress-strain curves for thin-film strips of PVA, 0.3 wt% BN/PVA, and 0.5 wt% BN/PVA are shown in Fig. 5h. By comparing with the parent PVA, the embedment of BN nanosheets in PVA resulted in a remarkable enhancement of ultimate tensile strength (194.8% increase) and Young’s modulus (307.4% increase). It is suggested that the mechanical strength of hybrid polymer films in comparison to the pure polymer matrix was significantly reinforced by loading rather modest weight fractions of BN nanosheets of 0.3 or 0.5 wt%^50^,^51^. In addition, in Fig. 5g, we also observed that light transmission properties of polymeric films did not change significantly after embedding BN nanosheets. BN nanosheets do not absorb in the visible region due to a wide band gap and thus have much less impact on the optical transmission of the polymer matrix compared to carbon fillers^52^.

## Discussion

In this study, we have established a versatile and efficient method for producing mono- and few-layered 2D nanosheets of graphene, MoS_2_, WS_2_, and BN, in the emulsions microenvironment of CO_2_/PVP/H_2_O system, and obtained the detail information about the optimal solution condition of their efficient exfoliation. Our study confirms that the driving forces originating from the phase inversion in the emulsion microenvironment efficiently facilitate the exfoliation of these layered materials, and the phase behaviours of micelles in the emulsions can be effectively manipulated by CO_2_ pressure and ethanol/water ratio as well. Subsequent experiment on the application of graphene demonstrates that the conductive films were successfully fabricated by this graphene ink and it exhibited the excellent conductivity. Application of exfoliated MoS_2_ on lung cancer cells indicates the broad-spectrum fluorescence labelling. Moreover, the exfoliated BN nanosheets were confirmed to be effective reinforcement on PVA films. Therefore it can be anticipated that this low-cost and environmental-friendly production of 2D nanosheets will supply a new platform to scale-up fabrication of more 2D layered nanomaterials and their heterojunction structure with excellent physical and chemical properties in the near future.

## Methods

### Exfoliation of layered materials

200 mg PVP (STREM CHEMICALS) were added into ethanol/water mixtures (10 mL) at an appropriate ethanol/water ratio. After PVP were completely dissolved, 50 mg powders of layered materials (Sigma Aldrich) were added to these solutions. Then these mixtures were sonicated in an ice bath for 30 min. These dispersions were quickly added into the supercritical CO_2_ apparatus composed mainly of a stainless steel autoclave (50 mL) with a heating jacket and a temperature controller. The autoclave was heated to 313.2 K, and then CO_2_ was charged into the autoclave to the desired pressure under stirring. After a reaction time of 3 h, the gas was released. Finally, the as-produced dispersions were sonicated in an ice bath for an additional 1 h, and then the dispersions were centrifuged at 3000 rpm for 20 minutes to remove aggregates, resulting in dark green solutions. The supernatants (top three quarters of the centrifuged dispersions) were collected by pipette.

### Materials characterization

UV-vis absorption spectroscopy was performed with a Shimadzu UV-240/PC with a scanning speed at 200 nm/min and a bandwidth 0.1 nm using the 1 cm quartz cuvette. Transmission electron microscope (TEM) images and associated electron diffraction (ED) patterns were taken with a JEOL JEM-2100 at an accelerating voltage of 200 kV. High resolution TEM (HRTEM) images were carried out with the JEOL JEM-2100 F, operated at 200 kV. The TEM and HRTEM samples were prepared by drying a drop of the suspension on a carbon film. Scanning electron microscopy (SEM) images were performed with the JEOR JSM-6700 F. A Digital Instruments Multi-Mode scanning probe microscope with a NanoScope V controller in tapping mode was used for the AFM measurements. The AFM samples were prepared by depositing a droplet of the suspension on Si/SiO_2_ substrate. Photoluminescence excitation spectra and fluorescence spectra were recorded with a Horiba Fluorolog-3 Fluorescence Spectrophotometer at room temperature. Fluorescence images were taken using the Olympus IX81 inverted research microscope equipped with the Olympus DP70 Color/Black and White camera (Olympus, America). An Olympus U-RFL-T power supply unit with a mercury lamp was used as the fluorescence light source. Cyclic voltammetry tests were carried out with the CHI 660D electrochemical workstation. The galvanostatic charge and discharge tests were carried out on a Neware battery tester (CT-3008) with a range of 0.005-3 V at different current densities. BN/PVA composites were mechanically characterised by tensile tester (UTM2203, Shenzhen Suns Technology Stock Co., Ltd, China) with a 100 N load cell at a strain rate of 5 mm min^−1^.

## Additional Information

**How to cite this article**: Wang, N. *et al*. High-efficiency exfoliation of layered materials into 2D nanosheets in switchable CO_2_/Surfactant/H_2_O system. *Sci. Rep*. **5**, 16764; doi: 10.1038/srep16764 (2015).

## Supplementary Material

Supplementary Information
