# Supplementary material for: High-efficiency exfoliation of layered materials into 2D nanosheets in switchable CO2/Surfactant/H2O system
Source: Sci Rep. 2015 Nov 16;5:16764. doi: 10.1038/srep16764 (PMC4645177; doi:10.1038/srep16764)
Supplement: Supplementary Information [file srep16764-s1.pdf]

High-efficiency exfoliation of layered materials into 2D nanosheets in switchable CO_2/Surfactant/H_2O system

Nan Wang[1], Qun Xu[1],* Shanshan Xu[1], Yuhang Qi[1], Meng Chen[1] and Hongxiang Li[1], & Buxing Han[2]

[1]College of Materials Science and Engineering, Zhengzhou University, Zhengzhou 450052, China.

[2]Institute of Chemistry, Chinese Academy of Science, Beijing 100080, China.

Correspondence and requests for materials should be addressed to Q.X. (email: qunxu@zzu.edu.cn).

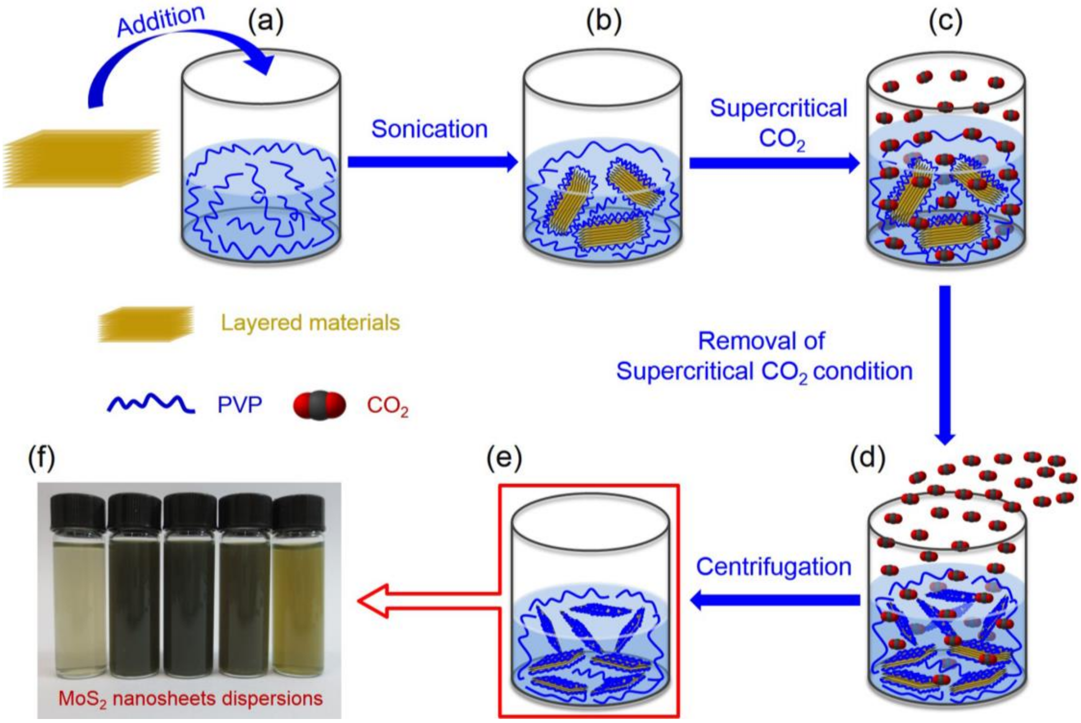

Supplementary Figure S1│ The schematic process to exfoliate layered bulk materials in the emulsions microenvironment of CO_2/PVP/H_2O system.

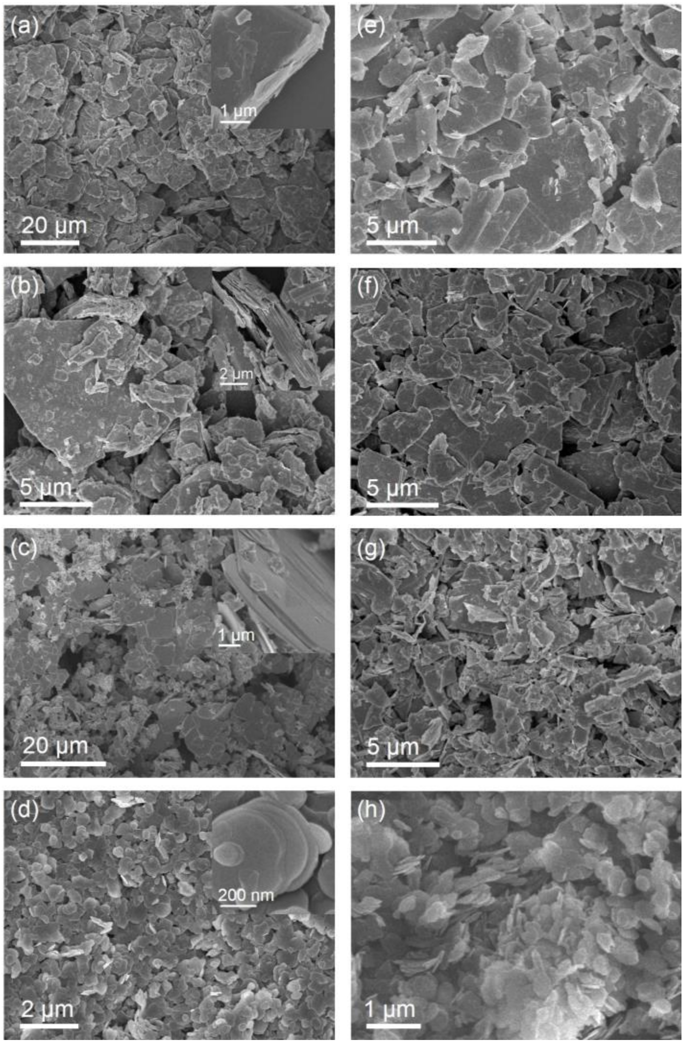

Supplementary Figure S2 │ SEM images of the starting powder of (a) graphene, (b) MoS_2, (c) WS_2, and (d) BN, and the sediment collected after centrifugation of (e) graphene, (f) MoS_2, (g) WS_2, and (h) BN.

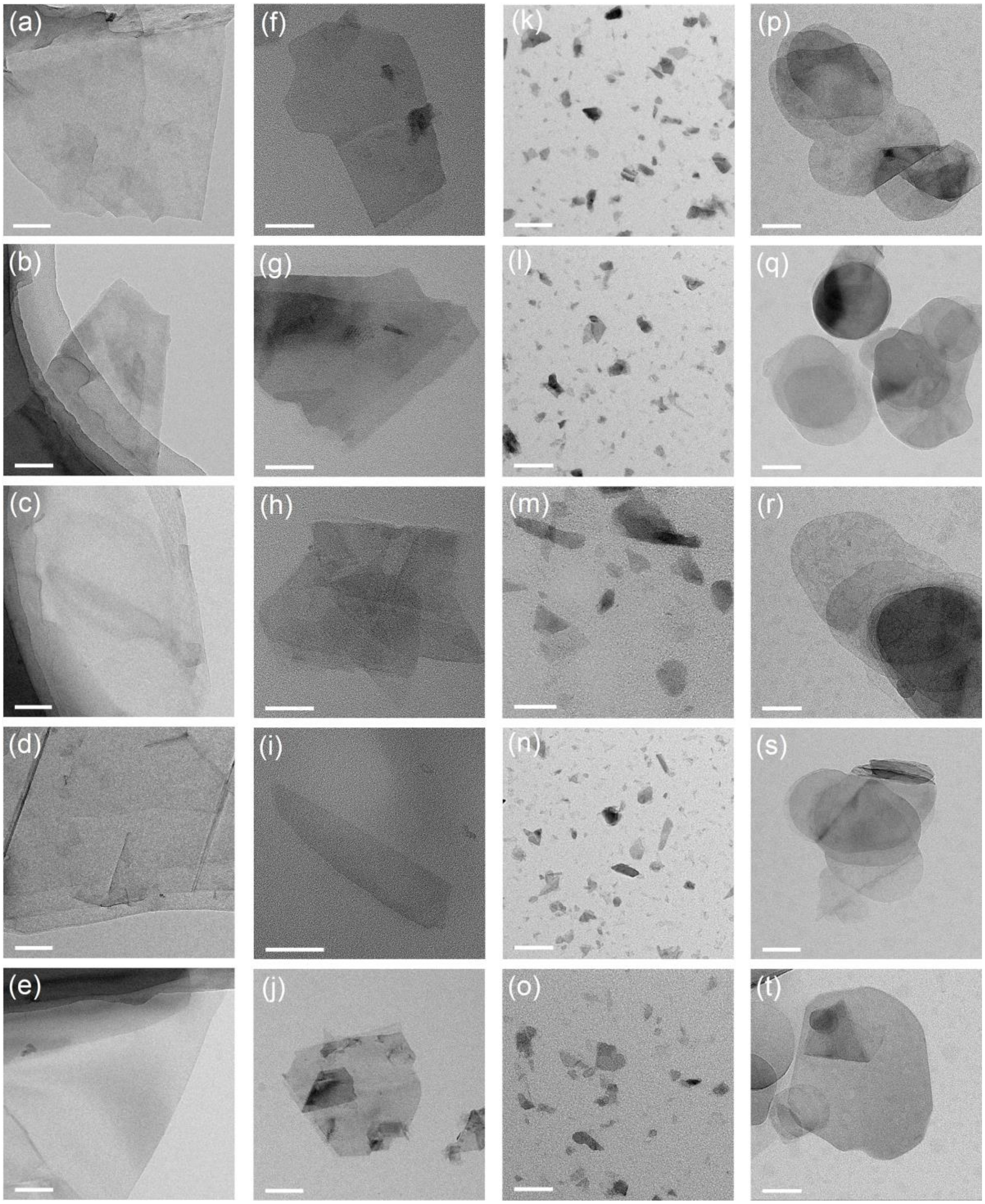

Supplementary Figure S3│TEM images of flakes of (a-e) graphene, (f-j) MoS_2, (k-o) WS_2, and (p-t) BN. In all cases the scale bar is 100 nm.

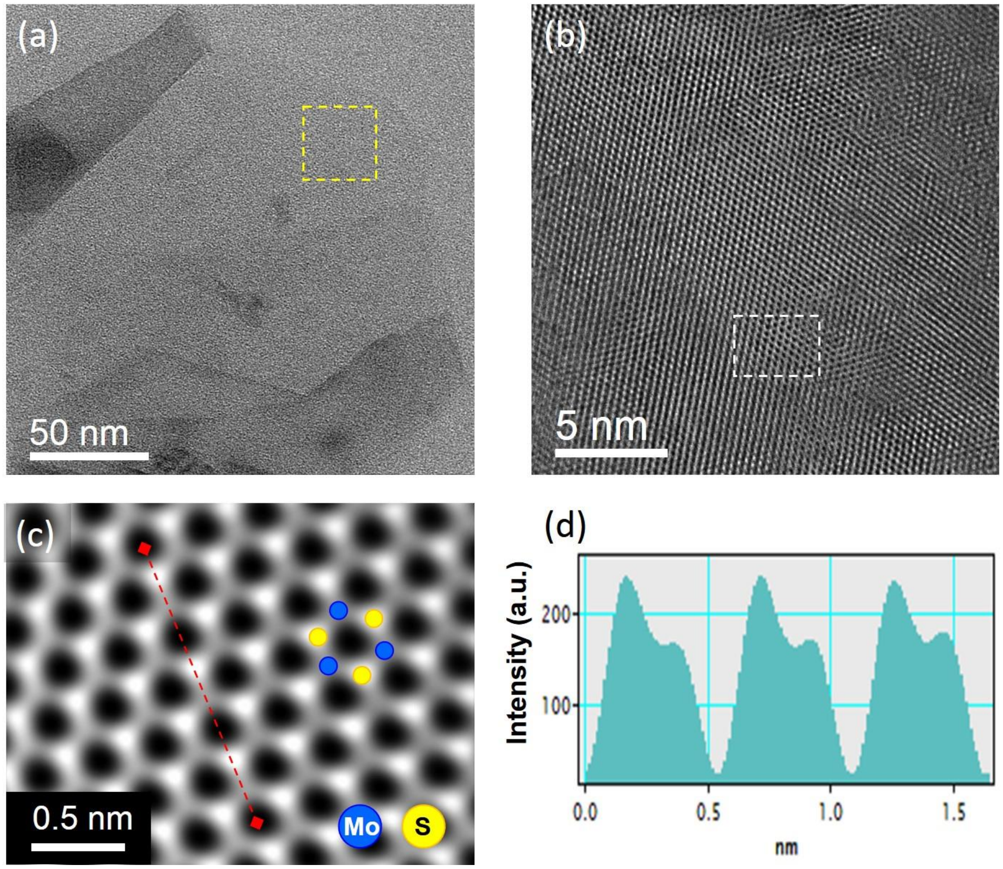

Supplementary Figure S4 | Identification of monolayer. (**a**) TEM images of a MoS_2 nanosheet in monolayer. (**b**) A HRTEM image of part of the region with monolayer enclosed by the yellow square of (a). (**c**) A filtered image of part of the region enclosed by the white square of (b). (**d**) Intensity distribution along the dotted line in (c) for the MoS_2 nanosheet.

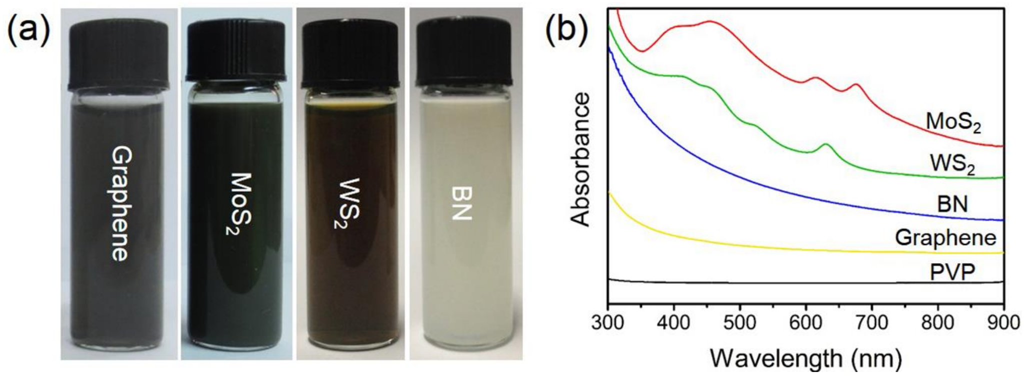

Supplementary Figure S5 | Optical characterization of exfoliated 2D nanosheets. (**a**) Photographs of dispersions of graphene, MoS_2, WS_2, and BN in ethanol/water mixtures, which have been stored under ambient conditions for two week. (**b**) Absorption spectra of dispersions of graphene, MoS_2, WS_2, BN, and pure PVP in ethanol/water mixtures. The absorbance value of pure PVP within the wavelength range of 300-900 nm is approximately zero, it means that the presence of PVP in the suspensions has no influences on the absorption spectra of these nanosheets.

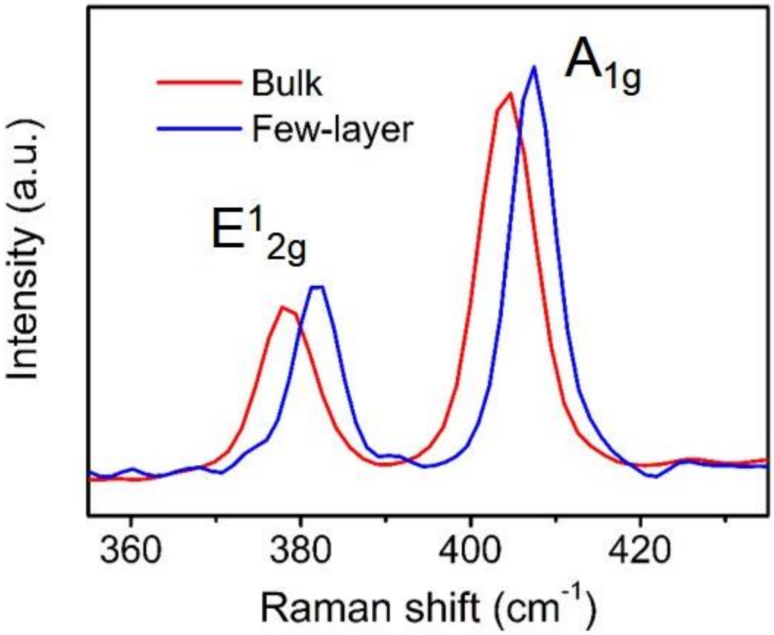

Supplementary Figure S6│Raman spectra of the few-layer and bulk MoS_2. The obtained few-layer MoS_2 presented two distinguished Raman features at 382.7 cm^{-1} and 407.4 cm^{-1}, which correspond to E^1_{2g} (in-plane vibration of two S atoms with respect to the Mo atom) and A_{1g} (out-of-plane vibration of S atoms) mode, respectively. Moreover, the deposited MoS_2 nanosheets show the distinct Raman fingerprint of the 2H-MoS_2 crystal with no evidence of structural distortion[25,43].

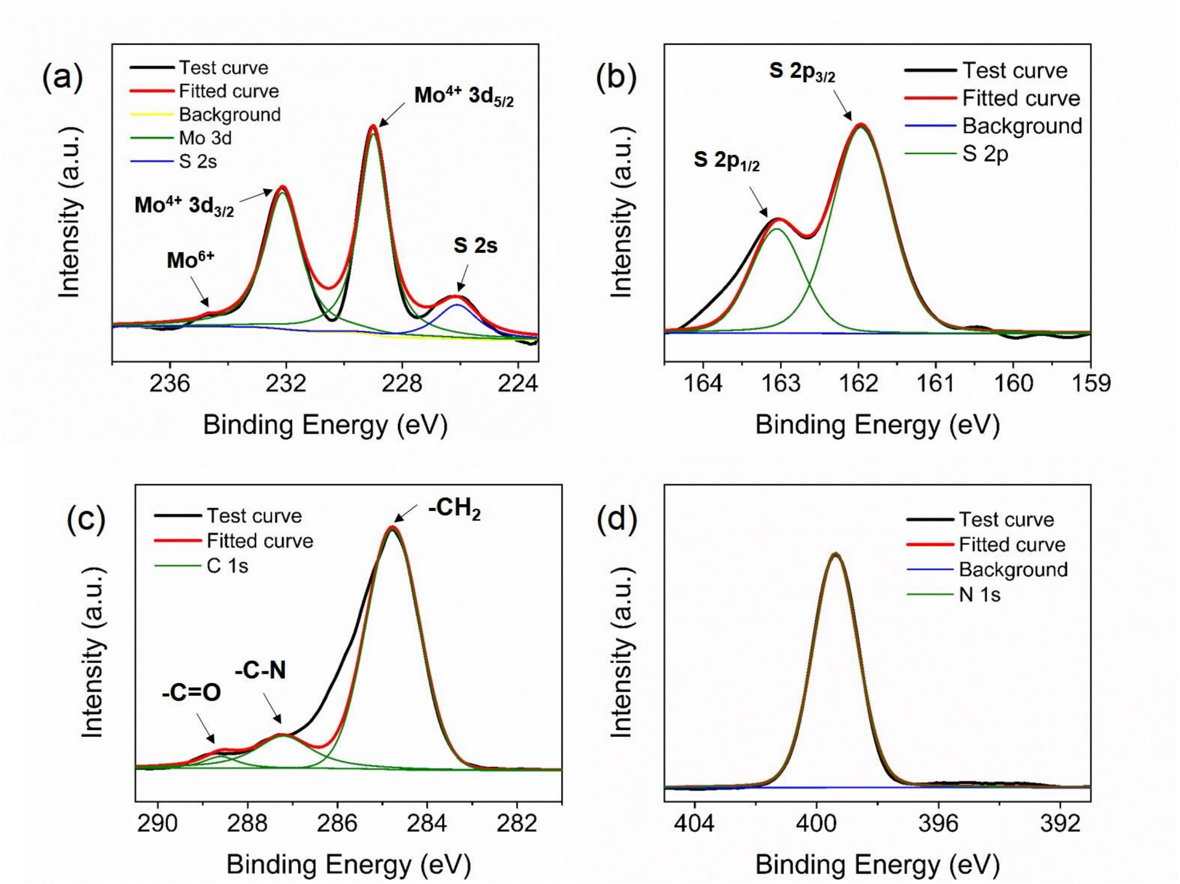

Supplementary Figure S7 | XPS spectra of MoS_2. (**a**) Mo 3*d* and (**b**) S 2*p* XPS spectra of exfoliated MoS_2. (**c**) C 1*s* and (**d**) N 1*s* XPS spectra of PVP adsorbing on the surface of MoS_2 nanosheets.

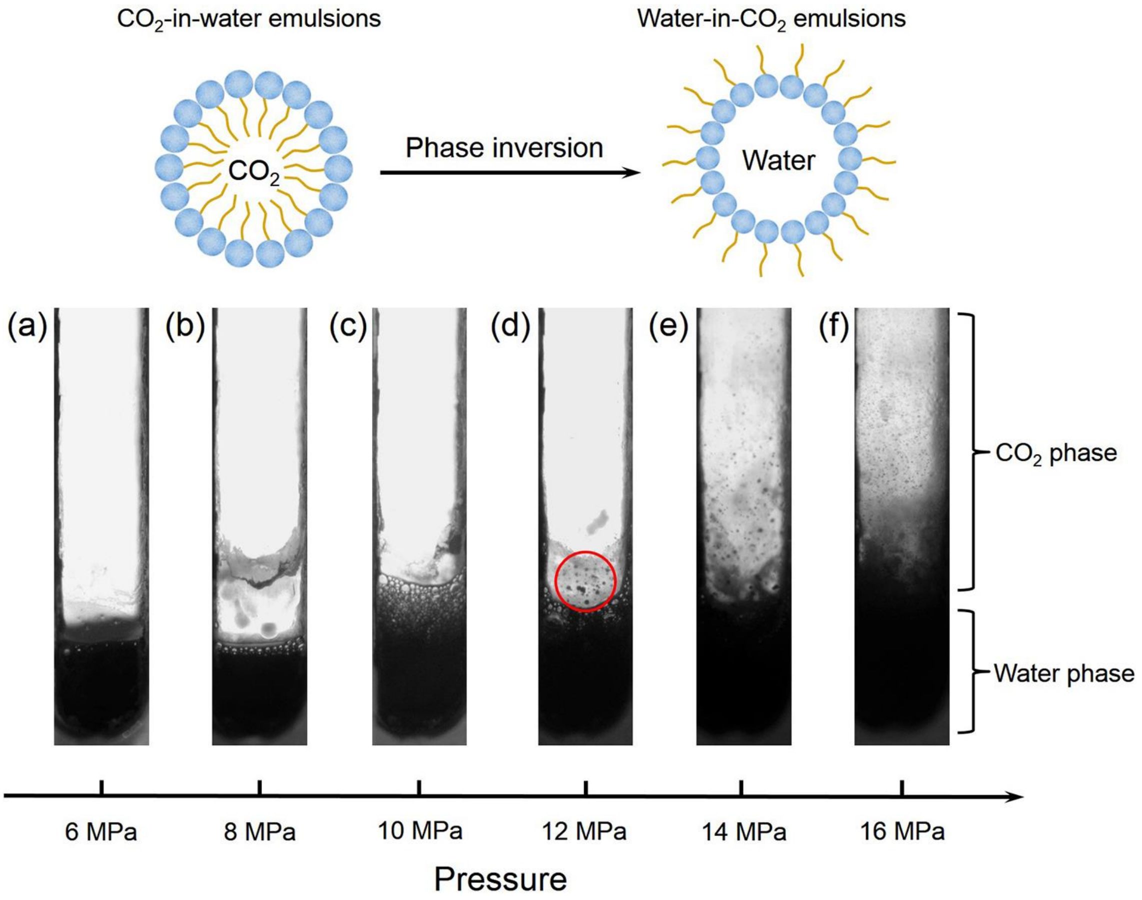

Supplementary Figure S8 | Phase behaviour of emulsions in the CO_2/PVP/H_2O system. Photographs of the CO_2/PVP/H_2O system with ethanol/water (ratio: 1:1) mixtures at 313.2 K and CO_2 pressures of: (a) 6 MPa, (b) 8 MPa, (c) 10MPa, (d) 12 MPa, (e) 14 MPa, and (f) 16 MPa.

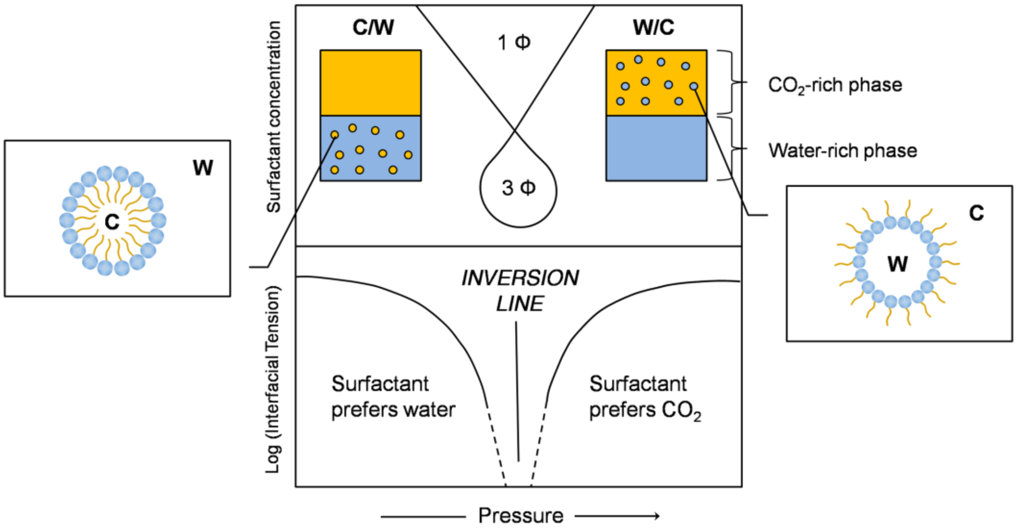

Supplementary Figure S9 | Schematic representation of the effect of CO_2 pressure or density on the phase behaviour and interfacial tension of CO_2/nonionic surfactant/H_2O system. W stands for water and C stands for CO_2.

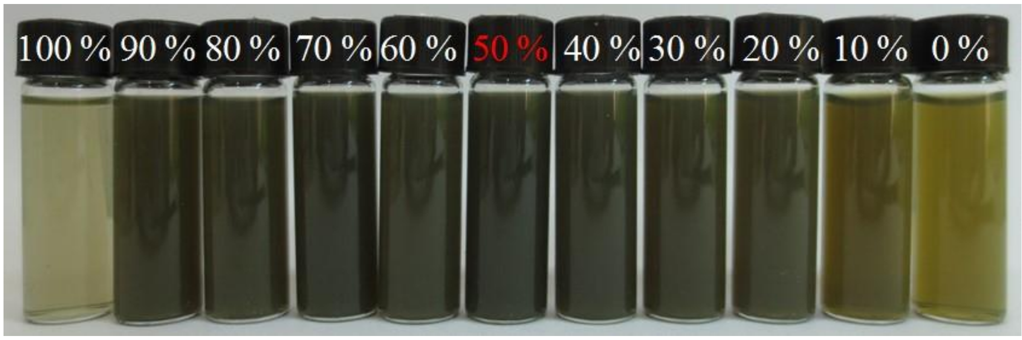

Supplementary Figure S10│Photographs of as-fabricated MoS_2 dispersions in ethanol/water mixtures with ethanol content ranging from 0 to 100 vol%.

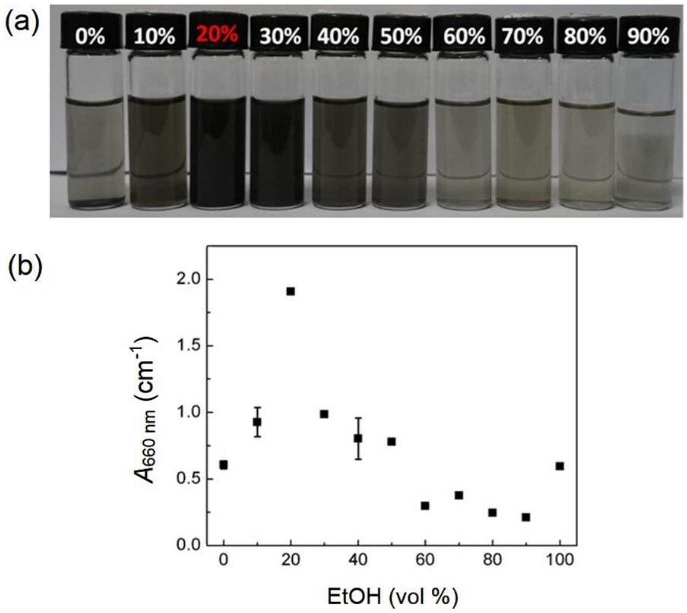

Supplementary Figure S11 | Photographs (a) and absorbance (b) at 660 nm of exfoliated graphene dispersions in ethanol/water mixtures with ethanol content ranging from 0 to 100 vol%.

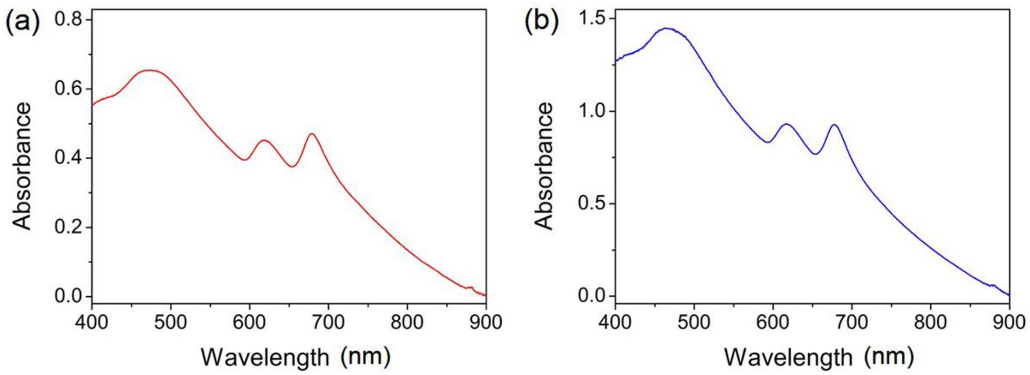

Supplementary Figure S12│The typical absorption spectra of exfoliated MoS_2 dispersed at ethanol/water (Ratio: 1:1) mixtures (a) with the absence of PVP and (b) without the assistance of supercritical CO_2.

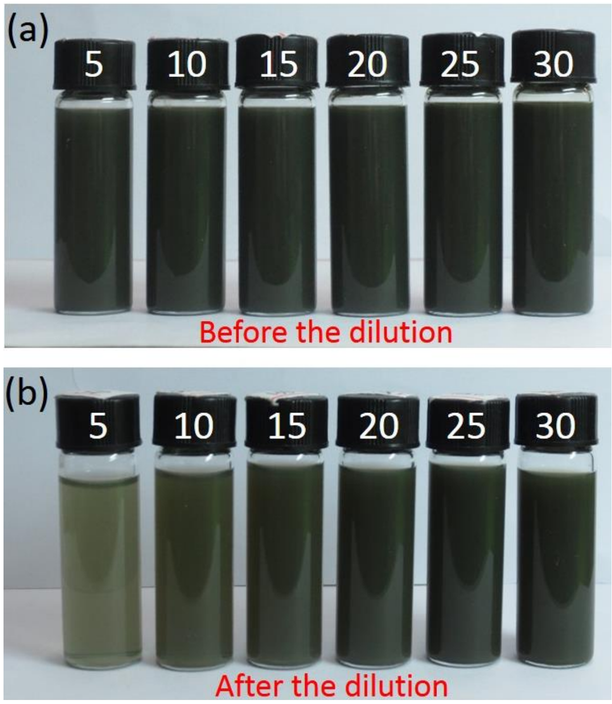

Supplementary Figure S13 | The relationship between exfoliated MoS_2 concentration (C) and initial MoS_2 concentration (C_I). (**a**) Photographs of dispersions containing MoS_2 nanosheets fabricated at different initial MoS_2 concentration (C_I) of 5, 10, 15, 20, 25, and 30 mg/mL. (**b**) Photographs of MoS_2 dispersions shown in (a) after five-fold dilutions.

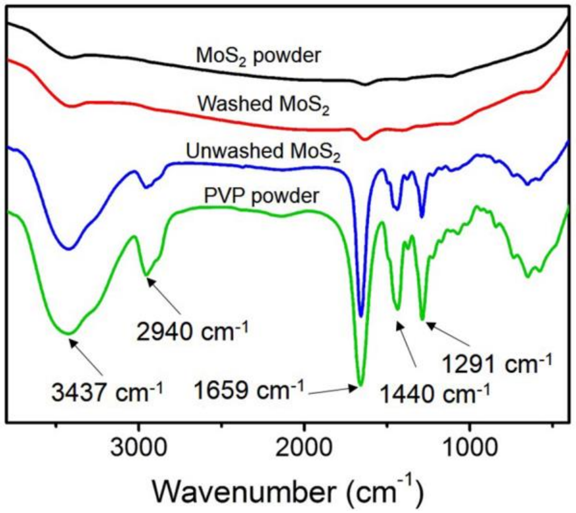

Supplementary Figure S14│FT-IR spectra of the starting MoS_2 powder, the MoS_2 nanosheets after washing off PVP, the MoS_2 nanosheets before washing off PVP and the starting PVP powder. The characteristic frequencies of the starting PVP powder can be clearly observed from the green curve. Concretely, the peak at 3437 cm^{-1} presents the typical stretching vibration band of O-H in water absorbed by PVP, the peak at 2940 cm^{-1} is due to the stretching vibration band of C-H, the peak at 1659 cm^{-1} is owing to the stretching vibration band of C=O, the peak at 1440 cm^{-1} is ascribed to the bending vibration bands of C-H, and the peak at 1291 cm^{-1} is assigned to the stretching vibration band of C-N. By observing the blue curve, we found that the unwashed MoS_2 nanosheets show the same characteristic frequencies as the starting PVP powder. Besides, the characteristic frequencies of washed MoS_2 nanosheets are consistent with those of the starting MoS_2 powder. Subsequently, these FT-IR spectra indicate that PVP absorbed on the surface of MoS_2 nanosheets can be washed away after exfoliation by centrifugation and filtration for many times.

Supplementary Tables

Supplementary Table 1. Concentration of the exfoliated 2D nanosheets dispersed in ethanol/water mixtures at different ratios.

Ethanol content / vol%	Absorbance (A_{672nm}) / cm^{-1}	Concentration / mg/mL^{-1}
0	0.582	0.038
10	0.769	0.051
20	1.090	0.072
30	1.328	0.088
40	1.324	0.087
50	2.102	0.139
60	1.166	0.077
70	1.542	0.102
80	0.977	0.065
90	0.947	0.063
100	0.477	0.032

Supplementary Note 1 Phase behaviour of emulsions in the CO_2/PVP/H_2O system.

In order to investigate the effects of emulsions environment of CO_2/PVP/H_2O system on the exfoliation, we observed the phase behaviour of the system at 313.2 K but with different CO_2 pressures (Supplementary Fig. S8). At low CO_2 pressure of 6 MPa, very small amounts of bubbles can be dissolved in water continuous phase, which is similar to that in the absence of CO_2. With the pressure of CO_2, an increasing number of bubbles appeared in water continuous phase, indicating formation of CO_2-in-water emulsions. When the CO_2 pressure reached 12 MPa, we found that some black droplets (marked by the red circle) appeared and floated in the CO_2 continuous phase. Besides, these water droplets were observed to settle over time, indicating water-in-CO_2 emulsions. Sedimentation is a result of the density difference between the water droplets and the CO_2 continuous phase. Subsequently, with continuous increase in pressure of CO_2, a growing number of black droplets existed in the CO_2 continuous phase and the size of black droplets became smaller and smaller based on the visual inspection. Because of the compressible nature of CO_2, an increase in pressure involves a reduction in the sedimentation velocity of water droplets in water-in-CO_2 emulsions. The phase behaviour of emulsions can be described in terms of mechanisms of phase inversion of emulsion as a function of formulation variables (see Supplementary Figure S9 and Supplementary Note 2)[41]. Usually the dynamic inversion of emulsions are strongly dependent on the phase behaviour, interfacial tension (γ), and emulsion curvature, which can be manipulated by varying a formulation variable, such as CO_2 density or pressure, ethanol/water ratio, and surfactant architecture (HCB).

Supplementary Note 2 Mechanisms of phase inversion of emulsions as a function of formulation variables

For supercritical CO_2 system, the dynamic inversion of emulsions is strongly dependent on the phase behaviour, interfacial tension (γ), and emulsion curvature. Moreover, the phase behaviour, interfacial tension (γ), and emulsion curvature depend on the interactions of the surfactant with each phase, which in turn determine where the surfactant partitions[42]. These items may be described in terms of the hydrophilic-CO_2-philic balance (HCB).

$$1/HCB = \frac{A_{TC} - A_{TT} - A_{CC}}{A_{HW} - A_{HH} - A_{WW}} \quad (1)$$

where A_{ij} is the interaction energy for the various interactions between CO_2 (C), surfactant tail (T), water (W), and surfactant headgroup (H). All interaction energies are written as positive values. This HCB is analogous to the well known hydrophilic-lipophilic balance (HLB) in traditional oil/water systems. The phase behaviour, interfacial tension (γ), and emulsion curvature, can be manipulated by varying a formulation variable, such as pressure, or ethanol/water ratio.

For CO_2/nonionic surfactant/H_2O systems, at low pressures, A_{TC} is small, the surfactant has a low tendency to partition toward CO_2. When the CO_2 pressure or density increases, 1/HCB increases (affinity toward CO_2) with CO_2 pressure or density. Increasing the CO_2 pressure or density at constant temperature increases the cohesive energy density of CO_2, resulting in an increase in A_{TC}. These reasons contribute to the partitioning of the surfactant toward the CO_2 phase, resulting in emulsion inversion from CO_2-in-water emulsions to water-in-CO_2 emulsions.

Supplementary Note 3 Graphene as electrode materials for supercapacitors

1. Preparation of graphene-ink and conductive paper

As-prepared graphene flakes were dispersed in DMF with the concentration of 10 mg/ml followed by sonication of 30 min. The resulting dispersion was applied to commercial paper with a paintbrush. Then the ‘wet’ paper was dried in the oven for 1 min at 120 °C. This simple ‘painting and drying’ process was repeated for a number of times to increase the graphene loading. The sheet resistance of the conductive paper was measured using a four-point probe system.

2. Fabrication of supercapacitors

For the laminated structure, two pieces of paper were used as both electrodes and were assembled into a supercapacitor by sandwiching a polypropylene (PP) membrane as the separator between the two electrodes and two pieces of stainless steel mesh without using conductive additives and binders. Cyclic voltammetry and the galvanostatic charge–discharge tests were carried out with the CHI 660D electrochemical workstation. The area capacitance value was calculated from cyclic voltammetry data according to the following equation:

$$C_{\mathrm{area}} = 2 \times \frac{\int I \mathrm{d}V}{A \times \Delta V \times \upsilon} \qquad (2)$$

Where C_{area} is the area capacitance based on graphene electrodes (F cm^{-2}), υ is the scan rate (V S^{-1}), $\int I\mathrm{d}V$ is the integrated area of the CV curve and A refers to the total area (cm^2) of the device.

Supplementary Note 4 MoS_2 Nanosheets as Fluorescent Label in Cellular Labeling

Two pieces of round cover glass (13 mm diameter, VWR International) were placed in a 12-well plate with 1 cover glass and 1 mL of DMEM culture medium per well. A total of 2×10^4 cells were plated in each well of that 12-well plate. The cells were incubated at 37 ℃ overnight to adhere on the cover glass. MoS_2 were added in culture medium, and then the cells were incubated at 37 ℃ for 24 h. After incubation, the cells on two cover glasses were washed with 1 mL of 1×PBS separately. Cells were fixed using 4 % paraformaldehyde at room temperature for 5 min. Then cells on cover glasses were washed with 1 mL of 1×PBS and mounted with Vectashield antifade mounting media. Cellular images were taken using the Olympus IX81 inverted research microscope equipped with the Olympus DP70 Color/Black and White camera (Olympus, America). An Olympus U-RFL-T power supply unit with a mercury lamp was used as the fluorescence light source.

Supplementary Note 5 BN as Fillers for the Mechanical Reinforcement of Polymer Films

BN nanosheets can be used as an excellent filler to prepare high performance polymer composites films. These nanosheets were then placed in vials of polyvinyl alcohol (PVA) dissolved in water at 20 mg/ml. The film mass and PVA/water volume were coordinated such that the BN nanosheets:PVA mass ratios were 0.3 wt% or 0.5 wt%. These dispersions were sonicated for 4 h to disperse the nanosheets in the PVA/water and then heated to 90℃ with gently stirring for 24h to ensure complete mixing. The resultant composite dispersions were then poured into a plastic mold and the solvent slowly evaporated. The resultant films were dried at 60℃ under vacuum for 48 hours (Figure 4G).

All free standing films and composites were cut into strips 10 mm wide and 25 mm long. These were mechanically characterised by tensile tester (UTM2203, Shenzhen Suns Technology Stock Co., Ltd, China) with a 100N load cell at a strain rate of 5 mm min^{-1}. Three and four strips were measured per sample for free standing films and composites respectively. All quoted numbers represent the average over the strips measured. Tensile testing results in stress strain curves such as those in Figure 5h,i. From these curves we can measure the Young's modulus (Y), the tensile strength (UTS), and the strain at break (ε).

Supplementary References

25. Liu, J. Q. *et al.* Preparation of MoS_2-polyvinylpyrrolidone nanocomposites for flexible nonvolatile rewritable memory devices with reduced graphene oxide electrodes. *Small* **8,** 3517-3522 (2012).

42. Lee, C. T., Jr., Psathas, P. A. & Johnston, K. P. Water-in-carbon dioxide emulsions: Formation and stability. *Langmuir* **15,** 6781-6791 (1999).

43. Wang, K. P. *et al.* Ultrafast saturable absorption of two-dimensional MoS_2 nanosheets. *ACS Nano* **7,** 9260-9267 (2013).
